# Effects of *Colocasia antiquorum* var. *Esculenta* Extract In Vitro and In Vivo against Periodontal Disease

**DOI:** 10.3390/medicina57101054

**Published:** 2021-10-02

**Authors:** Seong-Hee Moon, Seong-Jin Shin, Hyun-Jin Tae, Seung-Han Oh, Ji-Myung Bae

**Affiliations:** 1 Department of Dental Biomaterials and Institute of Biomaterials & Implant, College of Dentistry, Wonkwang University, 460 Iksan-daero, Iksan 54538, Korea; shmoon06@gmail.com (S.-H.M.); shoh@wku.ac.kr (S.-H.O.); 2 Department of Dental Biomaterials, College of Dentistry, Wonkwang University, 460 Iksan-daero, Iksan 54538, Korea; ko2742@naver.com; 3 College of Veterinary Medicine and Bio-Safety Research Institute, Jeonbuk National University, Iksan 54596, Korea; hjtae@jbnu.ac.kr

**Keywords:** *Porphyromonas gingivalis*, oral infection, periodontal diseases, oral microbiome, *Colocasia antiquorum*

## Abstract

*Background and Objectives*: Periodontal disease is a chronic inflammatory disease in which gradual destruction of tissues around teeth is caused by plaque formed by pathogenic bacteria. The purpose of this study was to evaluate the potential of 75% ethanol extract of *Colocasia antiquorum* var. *esculenta* (CA) as a prophylactic and improvement agent for periodontal disease in vitro and in vivo. *Materials and Methods*: The antimicrobial efficacy of CA against *Porphyromonas gingivalis* (*P. gingivalis*, ATCC 33277) was evaluated using minimum inhibitory concentration (MIC) and minimum bactericidal concentration (MBC) test, and cytotoxicity was confirmed by CCK-8 assay. For the in vivo study, *P. gingivalis* was applied by oral gavage to BALB/c mice. Forty-two days after the first inoculation of *P. gingivalis*, intraoral swabs were taken for microbiome analysis, and the mice were sacrificed to evaluate the alveolar bone loss. *Results*: The MIC of CA against *P. gingivalis* was 31.3 μg/mL, the MBC was 62.5 μg/mL, with no cytotoxicity. The diversity of the oral microbiome decreased in the positive control group, while those of the VA (varnish) and VCA (varnish added with CA) groups increased as much as in the negative control group, although the alveolar bone loss was not induced in the mouse model. *Conclusions*: CA showed antibacterial effects in vitro, and the VA and VCA groups exhibited increased diversity in the oral microbiome, suggesting that CA has potential for improving periodontal disease.

## 1. Introduction

Periodontal disease is a chronic inflammatory disease that causes gradual tissue destruction around teeth due to pathogenic bacteria [1]. Ultimately, severe periodontal disease leads to high mobility and loss of the tooth [2]. The oral cavity is a direct passage for micro-organisms to enter the body and a suitable habitat for various microbes [3]. Periodontal disease and associated oral pathogens, such as *Porphyromonas gingivalis* (*P. gingivalis*), have been epidemiologically and mechanistically associated with different systemic conditions such as coronary artery disease, rheumatoid arthritis, diabetes, and Alzheimer’s disease [4,5]. The prevalence and economic burden of periodontal disease will continue to increase as the aging population increases in size [6,7]. Therefore, the prevention and treatment of periodontal disease are essential for oral health and the overall health of adults. 

The oral microbiome represents microorganisms found in the human oral cavity [8]. If the balance of the oral microbiome is disturbed and harmful bacteria increase, it can cause systemic diseases, including periodontal disease [9]. Developing a dysbiotic microbiome characterized by increased total microbial load evokes gingival inflammation accompanied by the destruction of soft tissues [10]. Therefore, there has been ongoing interest in evaluating the composition and assembly of the oral microbiome associated with health and disease [11,12]. Due to the inherent simplicity of the mouse oral microbiome, the mouse model is worthy of investigation for understanding the mechanisms of human oral diseases such as periodontitis [11,13]. In addition, since the observed microbial genera in mice are often similar to the dominant genera found in humans, these animal models are also helpful in understaning the mechanisms of host–microbial interactions and homeostasis in health and disease [11]. 

Recently, natural products such as *Acacia arabica* [14], *Cymbopogam* [15], and *Magnolia officinalis* [16] have been used for the safe treatment of periodontitis, with fewer side effects than alternatives. In a previous study, the methanol extract of *Colocasia antiquorum* var. *esculenta* (CA) was confirmed to have antibacterial and anti-inflammatory effects and to inhibit osteoclast differentiation [17]. However, no studies have evaluated the efficacy of CA in vivo.

Therefore, we aimed to confirm the efficacy of 75% ethanolic extract of CA in vitro and in vivo. The antibacterial activities against *P. gingivalis* were measured in vitro. In vivo, oral microbiome and alveolar bone loss were evaluated in a *P. gingivalis*-induced periodontitis mouse model.

## 2. Materials and Methods

### 2.1. Preparation of Colocasia antiquorum var. Esculenta Extract (CA)

CA (Namwon-si, Korea) extract was purchased from the Korea Plant Extract Bank of the Korea Research Institute of Bioscience and Biotechnology (KRIBB, Daejeon, Korea). The method for preparing the extract was described in a previous study [17] except the extraction solvent was 75% ethyl alcohol. The plant was dried in the shade and powdered, and the powder was added to 1 L of ethyl alcohol 75% (GR Grade) and extracted through 30 cycles (40 KHz, 1500 W, 15 min ultrasonication, and 120 min standing per cycle) at room temperature using an ultrasonic extractor (SDN-900H, SD-ULTRASONIC CO., LTD., Seoul, Korea). After filtration and drying under reduced pressure, CA total extract was obtained with a yield of 4.74%. A stock solution of 50 mg/mL CA extract was prepared in dimethyl sulfoxide and stored at −20 °C until use.

### 2.2. Antibacterial Activity of CA

*Porphyromonas gingivalis* ATCC33277 was purchased from the Korean Collection for Type Cultures at the Korea Research Institute of Bioscience and Biotechnology (Jeongeup, Korea) and cultured anaerobically in brain heart infusion (BHI, BD, MD, USA) medium that included hemin (5 μg/mL) and menadione (0.5 μg/mL) at 37 °C. To test the antibacterial activity of CA, 100 μL of serially diluted CA at a concentration from 1.95 μg/mL up to 125 μg/mL was added to a 96-well plate. Then, 100 μL of *P. gingivalis* (1 × 10^5^ colony-forming units [CFU]/mL) was inoculated in each well. After 48 h, the minimum inhibitory concentration (MIC) of *P. gingivalis* growth was measured by optical density (OD) using a microplate reader (SpectraMax 250; Molecular Devices Co., San Jose, CA, USA) at a wavelength of 600 nm. For minimum bactericidal concentration (MBC) measurement, 100 μL of MIC samples from the MIC concentration to higher concentrations were inoculated on 5% sheep blood agar plates and incubated at 37 °C in anaerobic conditions for 7 days. The minimum concentration at which no growth of the *P. gingivalis* was observed was defined as MBC.

### 2.3. Cell Cytotoxicity Assay

Cell cytotoxicity was assessed using the CCK-8 assay kit (Dojindo, Molecular Technologies, Rockville, MD, USA). Then, 100 μL of L929 cells, the murine fibroblast cell line, were seeded into 96-well plates (1 × 10^5^ cells/mL) and incubated for 24 h at 37 °C in a humidified 5% CO_2_ incubator and treated with the same concentrations of CA as in the antibacterial assay. After 48 h, the supernatant was discarded and 100 μL of CCK-8 solution was added to each well and the plates were incubated for 1 h. The absorbance of the cultured cells was measured using a microplate reader (SpectraMax 250) at a wavelength of 450 nm, and the average value was calculated.

### 2.4. In Vivo Study Using a Mouse Model

**Figure 1:**
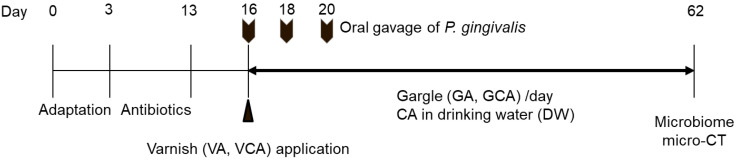
Experimental design of the mouse model according to time frame. After 3 days of adaptation, antibiotics were administered for 10 days followed by 3 days of antibiotic-free period. After oral gavage of *P. gingivalis* 3 times every 2 days, mice were treated according to group and sacrificed 42 days after the last inoculation of *P. gingivalis*. GA means gargle group: GCA, gargle containing *Colocasia antiquorum* var. *esculenta* (CA) group; VA, varnish application group; VCA, varnish with CA application group.

Ten-week-old female BALB/cAnNHsd (BALB/c) mice (20–25 g) were purchased from Koatech (Pyungtaek, Korea). The Institutional Animal Care and Use Committee of Wonkwang University approved all animal protocols (approval number: WKU20-17). All mice were housed at (22 ± 2) °C, humidity (55 ± 5)%, under a 12 h light/dark cycle, and received food and drinking water ad libitum. The mice were randomly divided into seven groups (n = 5/group) (Table 1). As shown in Figure 1, after adaptation for 3 days, the mice were treated with antibiotics (870 μg/mL sulfamethoxazole, 170 μg/mL trimethoprim) for 10 days in drinking water ad libitum to reduce the normal oral flora of all mice. Oral gavage of *P. gingivalis* in mice was performed as described previously [18]. Following a 3-day antibiotic-free period, 10^9^ CFU of *P. gingivalis* in 100 μL PBS with 2% carboxymethylcellulose (CMC; Sigma-Aldrich, St. Louis, MO, USA) was orally inoculated at 2-day intervals, up to a total of three times. For the NC group, 100 μL PBS with 2% CMC was administered. Two hours before the first inoculation, VA and VCA groups were anesthetized with urethane (Daejung, Siheung, Korea), and varnish was applied for 15 s to both maxillary molars. The varnish used in this study was described in a previous study [17]. In the VCA group, varnish was mixed with 15% CA. For GA and GCA groups, commercially available Garglin Zero™ (Dong-A Pharmaceutical Co., Ltd., Seoul, Korea) and 5% CA in Garglin Zero™, respectively, were sprayed at 50 μL orally once per day until the mice were sacrificed. The DW group received 1 *w*/*v*% CA in drinking water from the first oral infection to the end of the experiment. Forty-two days after the last inoculation, all mice were sacrificed to collect maxilla samples.

### 2.5. Oral Microbiome

For the collection of oral samples and extraction of genomic DNA from mice, a total of 22 samples were collected from each group using a swab analysis kit (Noble Bio, Hwaseong, Korea). A QIAamp DNA Stool Mini Kit (Qiagen, Hilden, Germany) was used to extract genomic DNA of bacteria in the oral cavity, and DNA was extracted according to the manufacturer’s protocol [19]. The library was prepared by referring to the 16S metagenomics library prep guide sequencing provided by Illumina, Inc. (San Diego, CA, USA). The clean-up process for the amplified product was performed using Agencourt AMPure XP beads (Beckman Coulter, Brea, CA, USA), and a library was produced using secondary PCR with an index. The library was quantified using Qubit 4.0 (Life Technologies, Stockholm, Sweden), and size was measured using Qsep1 (Bioptic Inc., New Taipei City, Taiwan). The concentration of the prepared library was adjusted to 20 pmol, and a mixture was made through pooling. Then, 20 μL of the sample was added to the cartridge, and the analysis was performed using the Iseq100 platform (Illumina). FASTQ files created by the Iseq100 equipment were analyzed to determine the metagenome on the EZBioCloud (ChunLab Inc., Seoul, Korea) platform. Operational taxonomic units (OTUs) were generated at a similarity level of 97%. The diversity and uniformity of microbial communities in the sample were analyzed using the Shannon diversity index and Simpson index.

### 2.6. Quantification of Alveolar Bone Loss by Micro-Computed Tomography (CT)

**Figure 2:**
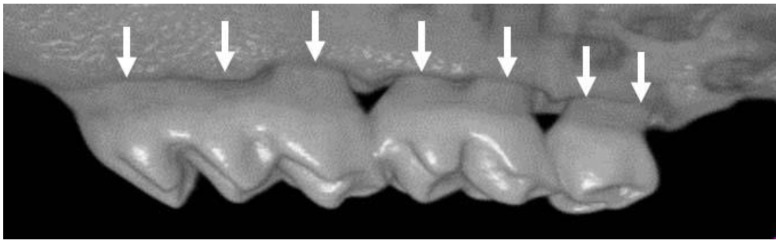
Measurement of alveolar bone loss as the linear distance between the cementum-enamel junction (CEJ) and the alveolar bone crest (ABC). Arrows indicate the measurement site.

Maxilla samples were fixed in 4% paraformaldehyde overnight and replaced with PBS. Micro-CT imaging was performed using a SkyScan 1076 microfocus X-ray system (Bruker, Kontich, Belgium) at a resolution of 9 μm as described previously [20]. All three spatial dimensions and each scan were reoriented using DataViewer (Bruker). For quantitative three-dimensional (3D) analysis of alveolar bone loss, 3D images were made using CTVox software (Bruker). The distance between the cementum-enamel junction (CEJ) and the alveolar bone crest (ABC) was measured with Image J as the average linear CEJ-ABC distance at a total of seven buccal sites per sample (Figure 2).

### 2.7. Statistical Analysis

All experiments were repeated at least three times. The statistical analysis was performed using SPSS (Statistical Package for Social Science, IBM Corp., Armonk, NY, USA) version 17, using non-paired *t*-tests and one-way analysis of variance (ANOVA) followed by multiple comparison tests. *p*-values < 0.05 were considered statistically significant.

## 3. Results

### 3.1. Antibacterial Activity of CA

**Figure 3:**
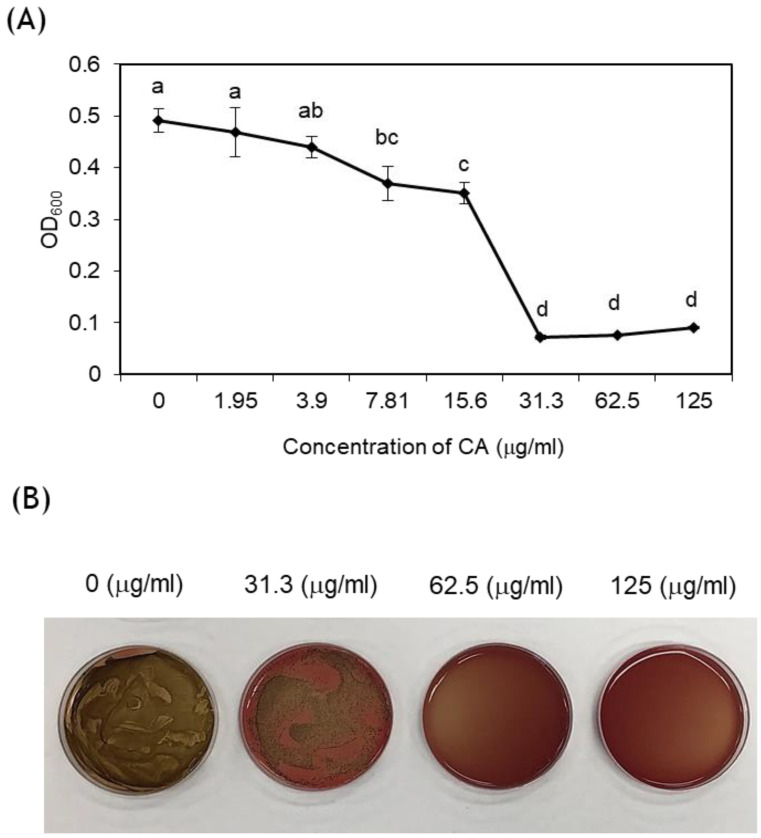
Antibacterial effects of *Colocasia antiquorum* var. *esculenta* extract (CA) treatments against *P. gingivalis*. (**A**) The minimum inhibitory concentration of CA. Different lowercase letters indicate significant differences between groups detected by one-way analysis of variance (ANOVA) with Tukey’s multiple comparisons (α ≤ 0.05). (**B**) The minimum bactericidal concentration of CA. At concentrations of 62.5 μg/mL or more, *P. gingivalis* did not form colonies.

Antibacterial activity of CA against *P. gingivalis* was indicated by MIC of 31.3 μg/mL (Figure 3A). The MBC of the CA was 62.5 μg/mL (Figure 3B).

### 3.2. Cell Viability of CA

**Figure 4:**
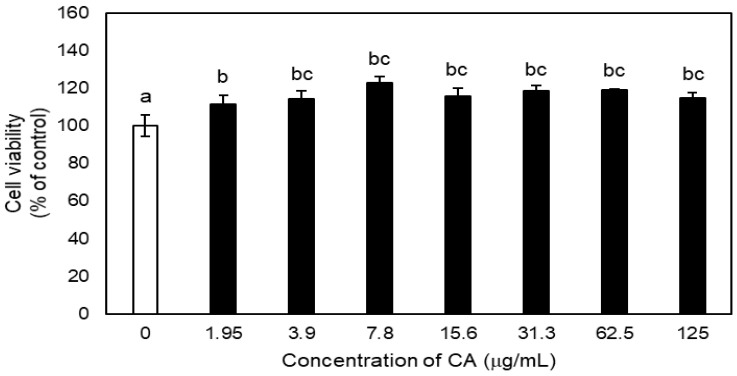
Effect of CA treatments on the cell viability of L929 cells depending on concentration measured by CCK-8 assay. Different lowercase letters indicate significant differences among groups detected by one-way ANOVA with Tukey’s multiple comparisons (α ≤ 0.05).

The cell viability of CA according to concentration is shown in Figure 4. There was no cytotoxicity compared to the control at any concentration of CA.

### 3.3. Microbiome Analysis

**Figure 5:**
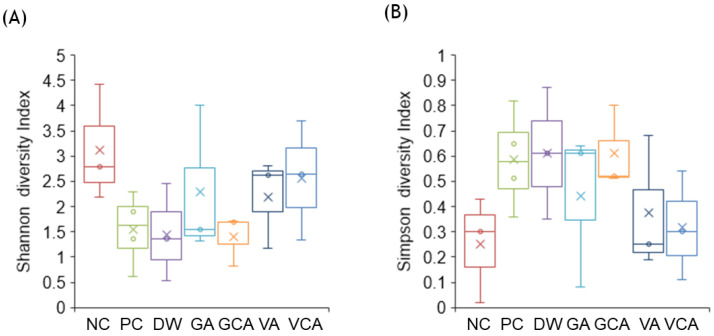
Alpha diversity of the oral microbiome. Boxplots show the Shannon diversity index (**A**) and the Simpson diversity index (**B**).

The mean of 82,298 sequences of each 16S rRNA gene was obtained from the samples. NC showed the highest diversity according to the Shannon diversity index, whereas the diversities of PC, DW, and GCA groups were low (Figure 5A). In contrast, the Simpson index, indicating evenness in the kinds of microorganisms present, showed high values in PC, DW, and GCA groups (Figure 5B). 

**Figure 6:**
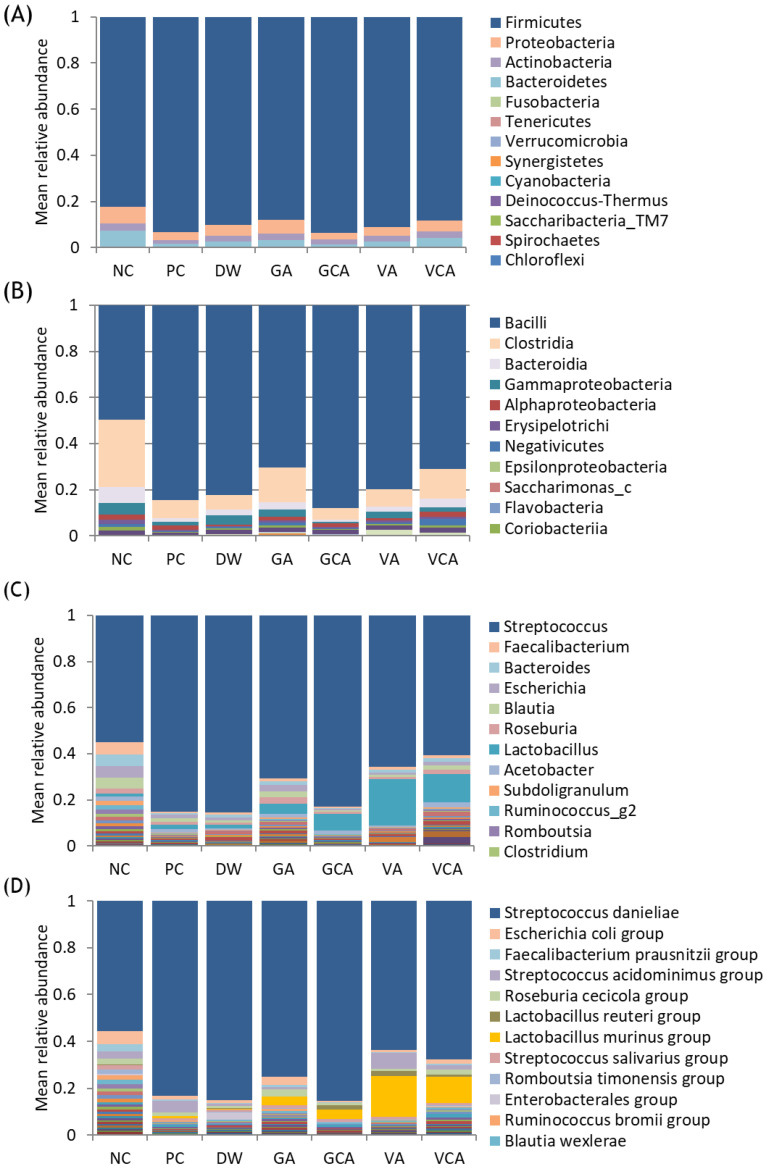
Bacterial community composition at the phylum (**A**), class (**B**), genus (**C**), and species levels (**D**). The mean relative abundance of each species detected in each sample is shown.

Bacterial distribution at the phylum level showed that Firmicutes was predominant, and Proteobacteria was the second most common (Figure 6A). Proteobacteria was the most common in the NC group but less common in the PC group, and tended to increase in all experimental groups except GCA. At the class level, Bacilli and Clostridia comprised 78–92% of the microbiome and *Bacilli* was predominant in PC, DW, and GCA groups (Figure 6B). Bacteroidia and Gammaproteobacteria were the third and fourth most common in NC, less common in PC, and increased in all experimental groups except GCA (Table 2). At the genus level, *Streptococcus* was predominant and in PC, DW, and GCA comprised 79–81% of the microbiome (Figure 6C). *Lactobacillus* was the second most common in all groups except NC and was found at higher frequencies in VA and VCA groups (Table 2). At the species level, a total of 2012 bacteria were detected with the most predominant species in all samples being *Streptococcus danieliae*, with highest frequencies in PC, DW, and GCA groups (Figure 6D). *Lactobacillus murinus* (*L. murinus*) was the second most common in GA, GCA, VA, and VCA groups, and higher in VA and VCA groups. *P. gingivalis* was more common in PC and GA groups (Table 2).

### 3.4. Alveolar Bone Loss

**Figure 7:**
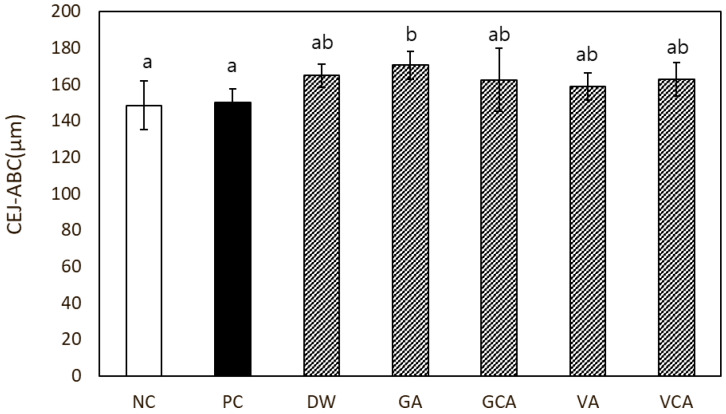
In vivo evaluation of alveolar bone loss in a mouse model. The amount of alveolar bone loss was measured by the distance between the cementum-enamel junction to the alveolar bone crest (CEJ-ABC). Different lowercase letters indicate significant differences among the groups by one-way ANOVA with Tukey’s multiple comparisons (α ≤ 0.05).

As measured the CEJ-ABC distance, the PC group did not show significantly higher alveolar bone loss compared to the NC group (Figure 7). 

## 4. Discussion

The effect of CA against periodontal disease was evaluated in vitro and in vivo. Antibacterial activity and cytotoxicity were evaluated using in vitro analysis, and the oral microbiome and alveolar bone loss were evaluated in vivo using a mouse model. We demonstrated that 75% ethanolic extract of CA has antibacterial activity against *P. gingivalis* 33277. In a previous study, the antibacterial activity of CA methanolic extract inhibited the growth of *P. gingivalis* by 40% [17]. We found that the MIC of 75% ethanolic extract of CA was lower than that of CA methanolic extract. Likewise, the MBC in the 75% ethanolic extract showed a lower concentration than in the methanolic extract. Therefore, the antibacterial effect of CA extract in 75% ethanol was more effective than CA methanolic extract. Moreover, the cell viability of CA was higher than that of the control group even at the effective antibacterial concentration. Therefore, 75% ethanolic extract of CA has an excellent antibacterial effect and high cell viability and can be used as a stable antibacterial agent.

Changes in diversity in the oral microbial community can be an index for evaluating periodontal disease because it is an essential indicator of many diseases, including tooth decay, periodontal disease, and systemic diseases such as diabetes, cardiovascular diseases, cerebrovascular diseases, atherosclerosis [9,21,22]. Whittaker has shown that diversity decreases as biome conditions become worse [23]. Disease states often lead to low diversity, making certain organisms better adapted to dominate their habitat, resulting in periodontitis and tooth decay [24]. In this study, the diversity of the microbiome decreased in the PC group compared with NC group. According to the Shannon index, the diversities of PC, DW, and GCA groups were low, consistent with groups with a dominant proportion of Bacilli at the class level, *Streptococcus* at the genus level, and *S. danieliae* at the species level. The presence of dominant bacteria indicates that other kinds of bacteria are reduced, resulting in the low diversity in the microbiome. Proteobacteria at the phylum level and Gammaproteobacteria at the class level are known to inhibit periodontal disease [1] and were most common in the NC group, but decreased in the PC group and tended to increase in all experimental groups except GCA. *Lacrobacillus* at the genus level and *L. murinus* at the species level were the second most common in VA and VCA groups. *Lacrobacillus* and *L. murinus* are considered to compete with the predominant bacteria. Thus, increasing amounts of *Lacrobacillus* and *L. murinus* could facilitate the diversification of the microbiome. The composition of the gargling agent (Garglin zero™) of GA and GCA groups was as follows: sodium fluoride, cetylpyridinium chloride (CPC), potassium sorbate, concentrated glycerin, saccharin sodium hydrate, citric acid monohydrate, purified water, and polyoxyl-40-hydrogenated caster oil. Among the compositions, there might be an antagonistic effect against CA. However, it is difficult to confirm which component of the gargle reacted with CA, because CA is the total extract of the plant, and the active ingredients are not specified yet. Interestingly, the VA and VCA groups, in which varnish or varnish with CA was applied to the teeth, only showed higher diversity according to the Shannon index than GA and GCA groups, in which gargling was performed on a daily basis, once. In the DW group, CA was supplied constantly in drinking water. Therefore, VA and VCA are considered to have the possibility to improve periodontal disease. 

As periodontitis progresses, the height of the alveolar bone gradually decreases, eventually resulting in tooth loss if not treated [25]. In this study, the amount of alveolar bone loss in the PC group was not significantly different from the NC group. This indicates that alveolar bone loss was not induced in the PC group. When periodontal disease is induced, changes in the oral microbiome occur first, followed by a gingival inflammatory response and finally alveolar bone loss [26,27,28]. In this study, the diversity of the oral microbiome decreased in the PC group, but bone loss did not occur. Therefore, the animal model in the study represented changes in the early stages of periodontal disease. The proportion of *P. gingivalis* detected was low, which means *P. gingivalis* did not predominate in the microbiome. The other potential reasons for the lack of difference in bone loss are as follows. First, the virulence of *P. gingivalis* is different depending on the strain. The virulence of *P. gingivalis* (ATCC 33277) used in the study is weaker than that of *P. gingivalis* (ATCC 53977, W50) [29]. Second, the amount of *P. gingivalis* may not have been sufficient for periodontitis induction, although we followed a previous protocol [30]. Because *P. gingivalis* is an anaerobic bacterium, survival will decrease when exposed to air as during the experiment. Thus, it is necessary to increase the amount of bacteria and the frequency of oral gavage. Third, according to Baker [30], the degree of bone loss due to periodontitis differs depending on the mouse strain. A/J, A/HeJ, 129/J, SJL/J, and C57BL/6J mice were more resistant to bone loss than were AKR/J, DBA/2J, BALB/cByJ, or BALB/cJ mice. In future studies, these variables should be considered to evaluate whether CA can inhibit bone loss. It is also necessary to confirm inflammatory changes. 

Our findings suggest that the 75% ethanol extract of *Colocasia antiquorum* var. *esculenta* exhibits antimicrobial activity without cytotoxicity in vitro. In addition, we found that the diversity of the oral microbiome was decreased in the PC group, and increased in the VA and VCA groups similar to the NC group. Therefore, CA is considered to have potential for preventing and treating periodontal disease when used with varnish. 

## 5. Conclusions

The results of this study suggest that the 75% ethanolic extract of *Colocasia antiquorum* var. *esculenta* exhibits antibacterial activity against *P. gingivalis* (ATCC 33277) and lacks cytotoxicity at the active concentration. The diversity of the microbiomes of CA-treated mice was high, suggesting potential applications for CA in the improvement of periodontal disease. 

## Figures and Tables

**Table 1:** Experimental groups in the animal study.

Group	Code	Treatment
Negative control	NC	Non-challenge with *P. gingivalis*
Positive control	PC	Oral challenge with *P. gingivalis*
CA in drinking water	DW	Oral challenge with *P. gingivalis*, 1 *w*/*v*% of CA in drinking water
Gargle	GA	Oral challenge with *P. gingivalis*, Garglin zero™
Gargle with CA	GCA	Oral challenge with *P. gingivalis*, 5% CA in Garglin zero™
Varnish	VA	Applied varnish, oral challenge with *P. gingivalis*
Varnish with CA	VCA	Applied varnish with 15% CA, oral challenge with *P. gingivalis*

**Table 2:** The proportions of bacteria expressed as means and standard deviations in parenthesis at the genus and species levels.

		NC	PC	DW	GA	GCA	VA	VCA
Class	Gammaproteobacteria	5.112 (1.507)	1.635 (1.009)	4.171 (3.714)	3.071 (2.711)	0.7697 (0.298)	2.875 (3.327)	1.871 (1.126)
Genus	*Lactobacillus*	1.027 (0.502)	1.866 (1.483)	1.623 (0.995)	4.131 (2.115)	7.021 (5.700)	17.821 (16.630)	11.131 (7.057)
Species	*L. murinus*	0.026 (0.021)	0.685 (0.883)	0.307 (0.341)	2.819 (2.965)	3.667 (3.014)	14.065 (19.074)	8.790 (7.304)
	*S. danieliae*	42.150 (31.206)	75.332 (13.578)	76.763 (17.017)	61.411 (30.131)	77.308 (10.311)	51.222 (29.464)	52.389 (19.910)
	*P. gingivalis*	0.001 (0.001)	0.013 (0.020)	0.000 (0.000)	0.011 (0.002)	0.005 (0.009)	0.001 (0.002)	0.006 (0.011)

NC, negative control; PC, positive control with *P. gingivalis* inoculation; GA, gargle group; GCA, gargle containing *Colocasia antiquorum* var. *esculenta* (CA) group; VA, varnish application group; VCA, varnish with CA application group.

## Data Availability

The data presented in this study are available on request from the corresponding author.
